# Sequence-Type Classification of Brain MRI for Acute Stroke Using a Self-Supervised Machine Learning Algorithm

**DOI:** 10.3390/diagnostics14010070

**Published:** 2023-12-27

**Authors:** Seongwon Na, Yousun Ko, Su Jung Ham, Yu Sub Sung, Mi-Hyun Kim, Youngbin Shin, Seung Chai Jung, Chung Ju, Byung Su Kim, Kyoungro Yoon, Kyung Won Kim

**Affiliations:** 1 Department of Computer Science and Engineering, Konkuk University, Seoul 05029, Republic of Korea; 87nasw@gmail.com; 2 Biomedical Research Center, Asan Institute for Life Sciences, Asan Medical Center, Seoul 05505, Republic of Korea; 3 Department of Radiology and Research Institute of Radiology, Asan Medical Center, University of Ulsan College of Medicine, Seoul 05505, Republic of Korea; ko.yousun82@gmail.com (Y.K.);; 4 Clinical Research Center, Asan Medical Center, Seoul 05505, Republic of Korea; 5 Department of Convergence Medicine, University of Ulsan College of Medicine, Seoul 05505, Republic of Korea; 6 Trialinformatics Inc., Seoul 05505, Republic of Korea; 7 Department of Radiation Science & Technology, Jeonbuk National University, Jeonju 56212, Republic of Korea; 8 Shin Poong Pharm. Co., Ltd., Seoul 06246, Republic of Korea; 9 Graduate School of Clinical Pharmacy, CHA University, Pocheon-si 11160, Republic of Korea; 10 Department of Smart ICT Convergence Engineering, Konkuk University, Seoul 05029, Republic of Korea

**Keywords:** magnetic resonance image, machine learning, metadata

## Abstract

We propose a self-supervised machine learning (ML) algorithm for sequence-type classification of brain MRI using a supervisory signal from DICOM metadata (i.e., a rule-based virtual label). A total of 1787 brain MRI datasets were constructed, including 1531 from hospitals and 256 from multi-center trial datasets. The ground truth (GT) was generated by two experienced image analysts and checked by a radiologist. An ML framework called ImageSort-net was developed using various features related to MRI acquisition parameters and used for training virtual labels and ML algorithms derived from rule-based labeling systems that act as labels for supervised learning. For the performance evaluation of ImageSort-net (ML_virtual_), we compare and analyze the performances of models trained with human expert labels (ML_humans_), using as a test set blank data that the rule-based labeling system failed to infer from each dataset. The performance of ImageSort-net (ML_virtual_) was comparable to that of ML_human_ (98.5% and 99%, respectively) in terms of overall accuracy when trained with hospital datasets. When trained with a relatively small multi-center trial dataset, the overall accuracy was relatively lower than that of ML_human_ (95.6% and 99.4%, respectively). After integrating the two datasets and re-training them, ML_virtual_ showed higher accuracy than ML_virtual_ trained only on multi-center datasets (95.6% and 99.7%, respectively). Additionally, the multi-center dataset inference performances after the re-training of ML_virtual_ and ML_humans_ were identical (99.7%). Training of ML algorithms based on rule-based virtual labels achieved high accuracy for sequence-type classification of brain MRI and enabled us to build a sustainable self-learning system.

## 1. Introduction

Recently, it has become common to collect multi-institutional data to develop and verify artificial intelligence in the field of medical imaging [1,2], and as available medical image data increases, curation work to refine and manage the data becomes more important [3,4]. However, medical imaging data has not been fully standardized, and the metadata of digital imaging and communications in medicine (DICOM) image files often differs across sites, vendors, and acquisition protocols [5,6].

Brain Magnetic Resonance Imaging (MRI) stands as an indispensable tool in the routine diagnosis and ongoing monitoring of individuals affected by various brain-related conditions, encompassing the realm of Brain-Computer Interface (BCI) technology. Its pivotal role lies in its ability to capture a diverse array of sequences, each offering a distinct set of data pertaining to anatomical structures, tissue density, and vascularization patterns within the brain. These sequences serve as a comprehensive means to portray both the anatomical nuances and pathological manifestations within the brain’s intricate landscape. Specifically, the multifaceted information derived from MRI sequences enables a detailed representation of the brain’s structural framework and provides invaluable insights into pathological alterations occurring within its complex network. Brain MRI, in particular, can acquire a variety of sequences, and each sequence provides these data [7].

In the field of stroke research, brain MRI is the most common imaging modality with various sequences, such as T1-weighted image (T1-WI), T2-weighted image (T2-WI), diffusion-weighted image (DWI), fluid-attenuated inversion recovery (FLAIR), perfusion images, susceptibility/gradient images (suscgre), and magnetic resonance angiography (MRA). It is crucial to sort brain MRI according to its specific sequence; however, owing to the variability of DICOM metadata, it is very time-consuming to classify brain MRI sequences according to the standardized sequence types [8]. Therefore, the need for automation for sequence-type classification of the brain MRI has been raised. Indeed, there have been several prior studies on automatic sequence types [9].

Current research on automatic sequence type classification has utilized supervised learning with manual labeling by human experts. However, manual labeling is a time-consuming and tedious task, which may hamper the updates to the automatic sequence type classification technique. A possible solution to this is self-supervised learning, which utilizes unlabeled data for training machine learning (ML) algorithms.

In self-supervised learning, an unlabeled training dataset can be labeled by using supervisory signals embedded in the data, such as metadata, or by using a pre-trained algorithm based on small amounts of human-labeled data [10,11]. Of these, the former method can be easily performed if there are established rules for labeling. In our imaging laboratory, we have established a rule-based labeling system for sequence type classification based on DICOM header information.

Thus, we aimed to develop a self-supervised ML algorithm for sequence-type classification of brain MRI for acute stroke using supervisory signals from DICOM metadata (i.e., rule-based virtual labeling).

## 2. Materials and Methods

This study was approved by the institutional review board of Asan Medical Center (AMC) (IRB No. 2022-0025). Because this is a retrospective study, informed consent was not required. The results in this study were reported according to the methods and terms in published literature guidance on ML for medical applications [12].

### 2.1. Data Sources and Dataset

A hospital and a multi-center trial dataset were used in this study (Table 1). For the hospital dataset, we collected from the picture archive and communication system (PACS) of AMC (PetaVision) consecutive brain MRI scans of 1528 patients (704 women; mean age, 59 ± 14.6 years) who underwent brain MRI for evaluation of stroke between January and February 2016. Given that AMC is a tertiary referral center, there were 357 MRI scans taken from lower-level hospitals in the PACS. We included such MRI scans with various manufacturers and sequences in the hospital dataset.

For the multi-center trial dataset, we used brain MRI scans that had been collected and archived for the clinical trial of SP-8203 (NCT 02787278) entitled “Safety and Efficacy of Two Doses of SP-8203 in Patients with Ischemic Stroke Requiring rtPA (SP-8203-2001)” [13]. The multi-center trial dataset contained 59 patients with 256 MRI scans from 8 medical centers in South Korea between June 2016 and August 2017. In the multi-center trial dataset, MRI scans were acquired from three different manufacturers (GE, Philips, and Siemens).

### 2.2. Generation of Human Expert Labeling

For the generation of human expert labeling (i.e., ground truth [GT]), we labeled eight MRI sequences that were commonly used for stroke evaluation, namely T1, T2, Scout, Diffusion, MRA, FLAIR, suscgre, and Perfusion. T1 refers to common T1-weighted sequences, including T1 spin echo (e.g., T1 SE), fast spin echo (e.g., T1 FSE), gradient (e.g., T1 MPRAGE), or FLAIR (e.g., T1-FLAIR), acquired by either a 2D or 3D scheme. T2 refers to common T2-weighted sequences, including 2D T2 fast spin-echo (e.g., T2 FSE) or 3D T2 fast spin-echo (e.g., T2 SPACE). Diffusion includes various kinds of diffusion-weighted images, including single-shot spin-echo echo-planar imaging (e.g., SE-EPI), multi-shot gradient echo-planar imaging (e.g., RESOLVE), or fast spin-echo imaging (e.g., FSE-DWI). MRA includes time-of-flight images (e.g., TOF-MRA) and contrast-enhanced MRA acquired by spoiled 3D T1 gradient echo sequences. FLAIR refers to the T2-FLAIR either in a 2D or 3D scheme. Suscgre includes susceptibility-weighted imaging acquired by a 3D gradient-echo sequence, echo-planar imaging, or 3D radial sequence, as well as T2*-weighted gradient-echo images. Perfusion includes both dynamic contrast-enhanced (DCE) and dynamic susceptibility contrast (DSC) imaging. All other types of sequences or images, such as MR spectroscopy and screenshots of the outputs of external post-processing software, were categorized as “Others” and excluded from the ML training.

The SeriesDescription and ProtocolName attributes were extracted from each MRI scan based on DICOM metadata attributes ((0008,103 E), (0018,1030), respectively). In each dataset, there were different values of SeriesDescriptions and ProtocolNames across different manufacturers and institutions, as presented in Supplementary Table S1.

Based on these attributes, the GT for the sequence types was generated by two experienced image analysts (S.J.H. and M.H.K.) and checked by a radiologist (K.W.K.). The total number of sequence types in the hospital dataset is presented in Supplementary Table S1. Results from the generation of human expert labels are described here as GT.

### 2.3. Development of a Rule-Based Labeling System

We developed a rule-based labeling system to generate virtual labels that can simulate and replace human expert labels. Rule-based labeling is a type of semantic-guided virtual label [14]. In addition, the rule-based labeling system is essential for advancing towards a sustainable artificial intelligence (AI) system [15].

Although the SeriesDescription and ProtocolName differ across manufacturers and institutions, they commonly include the same or similar keywords to indicate specific sequences. For example, the sequence type of diffusion-weighted image (hereafter referred to as diffusion) across many different SeriesDescriptions includes several common keywords, such as dw, dwi, diff, adc, and diffusion. We selected keywords that are commonly used in brain MRI for stroke so that they can be applied to data extracted from various hospitals and devices. We exclude ambiguous keywords that can be used in multiple sequence types. For example, the word maximum intensity projection (MIP) is used in the MRA sequence as well as other sequences, such as suscgre.

**Figure 1:**
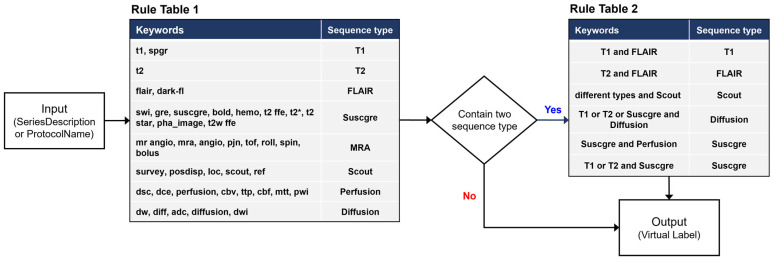
Rule-based labeling system overview for generating virtual labels.

The rule-based labeling system is composed of two steps (Figure 1). The first step is to identify predefined keywords in Rule Table 1 from the SeriesDescription attribute of the DICOM metadata of an MRI series. If the SeriesDescription attribute shows a null value (i.e., blank) or contains no predefined keywords, then the ProtocolName is searched. The second step is to determine the results. If there is only one keyword, the result is determined by the keyword. If there are two or more keywords in the same SeriesDescription or ProtocolName, a decision is made based on Rule Supplementary Table S1. For example, we classified T1-FLAIR as a T1 and T2-FLAIR as a FLAIR. Labels made through the rule-based labeling system are described here as virtual labels.

Our rule-based labeling system implemented under the above conditions may not cover sequence data with incomplete DICOM metadata. In such a case, the result of the rule-based labeling system is labeled as blank.

### 2.4. Design of ImageSort-Net Architecture

**Figure 2:**
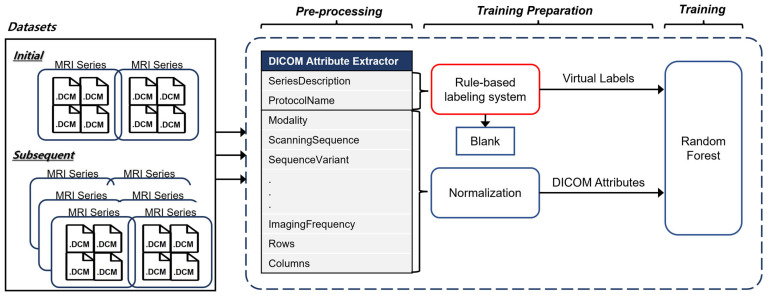
Overall steps to train ImageSort-Net and enable self-sustainable training.

Figure 2 illustrates the overall architecture of our ImageSort-net, which classifies the MRI sequence type. Our model is developed as a self-sustainable MRI sequence-type classifier algorithm, composed of three steps as follows:

(1) The pre-processing step extracts various DICOM metadata, including the SeriesDescription attribute, the ProtocolName attribute, and many other attributes associated with MR acquisition parameters.
(2) Training preparation step, which prepares necessary data for supervised learning of the ML algorithm, including virtual labels derived from our rule-based labeling system and features derived from normalization of DICOM attributes.
(3) Training step that trains the random forest ML algorithm.

### 2.5. Pre-Processing Step

In this step, various DICOM metadata, including the SeriesDescription attribute, the ProtocolName attribute, and many other attributes associated with MR acquisition parameters (such as RepetitionTime, EchoTime, and FlipAngle), were extracted. To reduce pre-processing step time, we extracted only information necessary for the rule-based labeling system and ML algorithm training instead of extracting all DICOM attributes (Table 2). The DICOM attributes are extracted in each series of an MRI scan. We additionally obtained the number of slices, not directly derived from the DICOM property, per series using the SeriesInstanceUID (0020,000 E). We used the Python Pydicom package.

### 2.6. Training Preparation Step

In this step, ImageSort-net prepares virtual labels derived from our rule-based labeling system, which work as labels for supervised learning. Moreover, it prepares various features associated with MRI acquisition parameters for the training of the ML algorithm. These features were selected by two MRI experts (K.W.K., S.C.J.). The normalization of selected DICOM attributes is performed to change the values of numeric columns in the dataset to a common scale without distorting differences in the ranges of values; this is helpful in providing features for ML algorithm training.

### 2.7. Training Step

**Figure 3:**
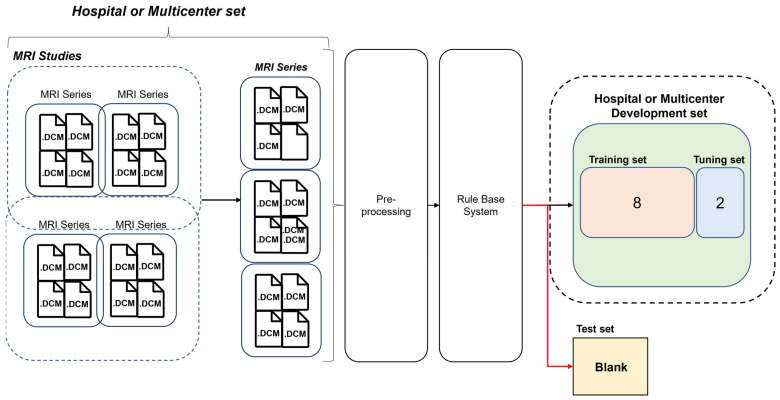
Development and test set details for the hospital and multi-center trial datasets.

The development dataset of ML algorithms was constructed except for blank data that the rule-based labeling system fails (by design) to infer from the dataset. And since the blank data were not used during learning, it is suitable for use as a test dataset. We evaluate and analyze two models learned with human expert labels (ML_human_) and rule-based virtual labeling (ML_virtual_) using a blank dataset as a test set (Figure 3).

We chose a random forest classifier for this study, with the random forest algorithm still being the most commonly used method with proven robustness to overfitting. This classifier enables easy handling of missing values and is effective for large dataset processing owing to its fast training and testing speed. Therefore, it is suitable for the MRI header data processing that we are dealing with.

**Figure 4:**
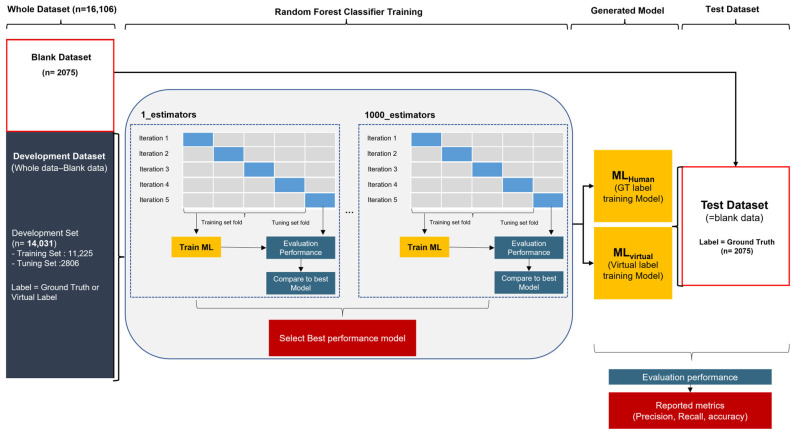
Process of training and evaluating random forest classification algorithms with hospital datasets.

We used the scikit-learn application programming interface (API) for ML for the random forest classifier, and we set all parameters to default values, except the estimator. The estimator is a variable that determines the number of decision trees in a random forest and is an important parameter in relation to overfitting. To find the optimal hyperparameter, we generated 1 to 1000 estimators, conducted repeated experiments, and used 5-fold cross-validation for each training. In cross-validation, the development set is randomly divided into five groups. Each group is used as a validation set at least once for five iterations, and the remaining is used as a training set. Details of the ML learning and evaluation are shown in Figure 4.

### 2.8. Classification and Performance Evaluation

Using the hospital and multi-center trial datasets, the performances of the rule-based labeling, ML_virtual_, and human-expert ML_human_ algorithms in classifying MRI sequences were evaluated. For performance measurements, we used the human expert labeling of sequence types as a reference standard.

Classification performance on total sequences (i.e., the sum of all sequences) was evaluated using overall accuracy, and classification performance on each sequence type was evaluated using F1 scores, precision, and recall. The F1-score, precision, recall, and accuracy were defined as:

(a) F1-score = 2 × (presicion × recall) ÷ (precision + Recall)
(b) Precision = TP/(TP + FP)
(c) Recall = TP/(TP + FN)
(d) Overall accuracy = (TP + TN)/(TP + TN + FP + FN)

### 2.9. Feasibility Experiment for Sustainable Self-Learning M_Lvirtual_ Algorithm

The concept of a sustainable self-learning ML_virtual_ algorithm is to add new datasets to the preexisting datasets and train the ML algorithm again to increase its classification accuracy. Thus, we performed an experiment to add the multi-center trial dataset to the hospital dataset and re-trained the ML_virtual_ and ML_human_ algorithms using the combined dataset. The training of ML_virtual_ and ML_human_ with the multi-center trial dataset was performed for comparison. Subsequently, the re-training using the combined dataset (hospital dataset + multi-center trial dataset) was performed by following the previously outlined three steps: pre-processing, preparation, and training steps, as illustrated in Figure 2. In the re-training, there was no need for human labeling, given that ML_virtual_ is an automatic self-learning system. The performance of the ML algorithms was compared between the hospital dataset, the multi-center trial dataset, and the combined dataset (hospital dataset + multi-center trial dataset).

## 3. Results

### 3.1. Classification Performance of a Rule-Based Labeling System

Table 3 presents the MRI sequence type classification performance of the rule-based labeling system, which does not use ML. Each sequence classification performance on the hospital and multi-center trial datasets were generally good, with F1-scores ranging from 92.5% to 100% (median 99.0%). Only the Perfusion sequence type showed relatively low F1-scores (92.5%) with the multi-center dataset, while the other sequence types showed high accuracy (94.5–100%). The associated confusion matrix can be found in Supplementary Figure S1.

There were 2075 (12.9%) blank cases for the hospital dataset and 347 (11%) blank cases for the multi-center trial dataset, in which a sequence type cannot be inferred from a rule-based labeling system.

### 3.2. Classification Performance of the Initial ML Algorithm on the Hospital Dataset

Table 4 demonstrates the performance of the initial ML_virtual_ and ML_human_ algorithms to classify MRI sequence types in the hospital dataset. In total sequence type classification, the overall accuracy of the ML_virtual_ algorithm was comparable to that of the ML_human_ algorithm (98.5% and 99%, respectively). In each sequence type classification, the F1-score of ML_virtual_ for Scout was relatively low (50%) compared to other sequence types (ranging from 80% to 100%, respectively) (Table 5), which might be attributed to the substantial heterogeneity across vendors and institutions in the DICOM metadata as well as in acquisition parameters. Supplementary Figure S2 shows the confusion matrix associated with the performance of both ML models.

### 3.3. Classification Performance of Subsequent ML Algorithms with Additional Dataset

The classification performances of ML_virtual_ and ML_human_ trained only on subsequent datasets (multi-center trial datasets) are presented in Table 4. The overall accuracy of the ML_virtual_ algorithm in total sequence type classification was relatively lower than that of the ML_human_ algorithm (95.6% and 99.4%, respectively). In each sequence classification, the F1-score of ML_virtual_ for suscgre was relatively low (52.6%) compared to other sequence types (ranging from 97.3% to 100%, respectively) (Table 5). Supplementary Figure S3 shows the confusion matrix associated with the performance of both ML models.

After integrating the hospital dataset and multi-center trial dataset into a large, combined dataset, the re-trained ML_virtual_ model following its sustainable self-learning scheme showed better overall accuracy (99.7%) compared to that with the multi-center trial dataset alone (95.6%), as presented in Table 4. Furthermore, the inference performance on multi-center datasets of ML_virtual_ and ML_human_ after re-training was the same. Supplementary Figure S4 shows the confusion matrix associated with the performance of both ML models. These results indicate that sustainable self-learning ML algorithms using rule-based virtual labeling in the new datasets are feasible.

### 3.4. Clinical Effectiveness of ImageSort-Net

**Figure 5:**
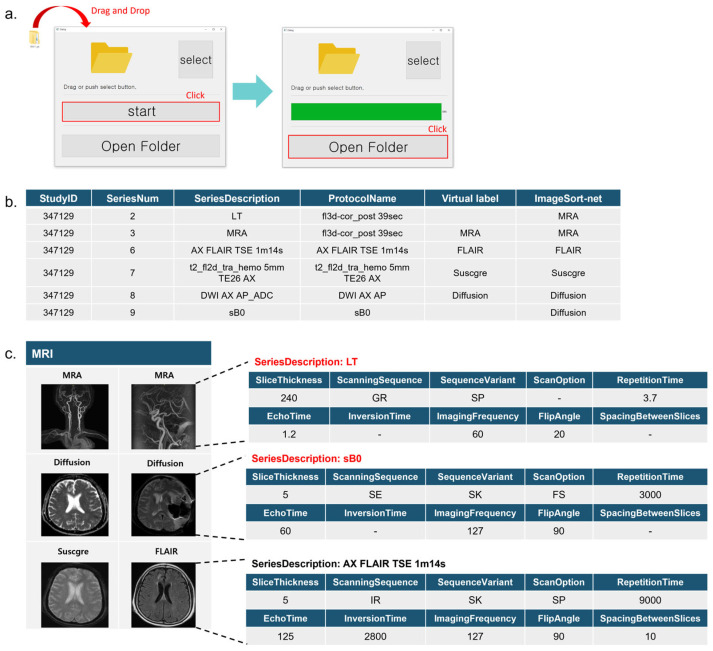
ImageSort-net GUI software. (**a**). ImageSort-net GUI software. (**b**). Sample of ImageSort-net results for one MRI scan. (**c**). Incomplete header data were accurately inferred from ImageSort-net.

We applied ImageSort-net in our clinical trial imaging core lab (Asan Image Metrics, www.aim-aicro.com (accessed on 20 November 2023)) in the form of a graphic user interface (GUI) software (v1.0.0) tool (Figure 5), which greatly saved time for image analysts to label MRI sequences. Indeed, the multi-center trial datasets (256 MRI scans with 3146 sequences) were manually labeled by an image analyst (M.H.K. with 15 years of experience in MRI research), which took seven full working days (8 h per day). On the contrary, the actual time spent by ImageSort-net for the overall classification of 256 MRI scans was 305 s for only inference and approximately 3 h for training.

## 4. Discussion

We created a rule-based labeling system using the metadata of DICOM image files and developed a sustainable self-supervised ML algorithm, named ImageSort-net, for automatic sequence-type classification of brain MRI using supervisory signals from DICOM metadata (i.e., rule-based virtual labeling).

Our rule-based labeling system achieves high performance with an overall accuracy of 99.5% as long as DICOM metadata is enough for classification. However, if the header is empty or the keyword is not in the rule, the rule-based labeling system cannot classify the sequence type and leaves the blanks, which account for more than 11% of the dataset. These blanks were inferred by the ML model in the ImageSort-net, and it achieved high classification performance with an overall accuracy of 98.6%.

Our motive for developing sustainable self-learning machine learning was to reduce the need for human resources and time in the classification of MRI sequences in multi-center clinical trials for acute stroke. Owing to the variability of DICOM metadata, it is very time-consuming to classify imaging sequences according to the standardized sequence types [16]. ImageSort-net can save time and cost by replacing labeling tasks in clinical trials and solving cases where manual labeling may vary depending on the experience of radiologists. In the MRI series of approximately 20,000 used in this study, the virtual labeling process takes 2 to 3 h, which is a task that takes weeks to months if carried out manually. Our proposed algorithm can replace the time-consuming data preparation process in image-based AI algorithm development.

Recently, research on the automatic classification of MRI series has been actively conducted, mainly based on images and text [17]. The image-based deep learning model classifies brain MRI series into eight classes with a high overall accuracy of 98.5% and uses a structure that sorts data according to predictive results. However, the data preparation process for learning early CNNs is essential, and manual labeling is required for model change [18]. For text-based ML models, construct a large dataset (700,000 MRI series) and develop it based on DICOM headers. Approximately 80% of the data sets were labeled based on the Series Description, while the remaining 20% were labeled after image verification. Model accuracy achieved high performance (97.4% to 99.96%).

Compared to these prior studies requiring manual labeling, we propose an MRI series classification system that does not require human interference for data extraction and allows learning by replacing labeling tasks with a rule-based labeling system, achieving performance comparable to models learned by manual labels. We developed ImageSort-net using approximately 20,000 MRI series, but its performance closely approximates that of models using 700,000 MRI series (98.6% and 99.6%, respectively).

In addition, ImageSort-net showed reliable performance by appending a new dataset to an existing dataset without human labeling of the combined dataset. Enhancing external validity is crucial to ensuring the robustness and adaptability of diverse imaging acquisition protocols across different manufacturers. We believe that external validity can be effectively achieved through a retraining process. Our findings, illustrated in Table 5, demonstrated consistent performance when incorporating a new dataset into an existing one without the need for human labeling of the combined dataset. These results underscore the capability of achieving robustness and adaptability to varied imaging conditions through retraining methods. Typically, retraining procedures can be laborious and challenging. However, our proposed algorithm significantly simplifies this process by eliminating the need for manual intervention. This innovation enables us to conduct retraining seamlessly, enhancing ease of implementation and reducing the associated complexities. Consequently, our result indicates that sustainable self-learning ML algorithms using rule-based virtual labeling in new datasets are feasible.

In addition to the automatic sequence type classification, it is also very important to provide detailed information such as the 2D/3D scheme, image plane, and parameter characteristics. For automatic sequence type classification, machine learning is very useful. For instance, we intend to categorize various kinds of MRA images, including raw images (mask) and reconstructed MIP or rotation images, as the “MRA” sequence type. Additionally, for diffusion-weighted images, our algorithm categorizes b0 images, b1000 images, and ADC images as the “DWI” sequence type. For the detailed information, we can extract it directly from the DICOM metadata (i.e., attributes) rather than using machine learning. For example, the DICOM attribute (0018,0023) includes the spatial data encoding scheme, which can be either 2D or 3D. The DICOM attribute (0020,0037) specifies the plane of acquisition for an image, either axial, sagittal, or coronal. In the DWI, detailed information such as the diffusion b-value can be extracted from the DICOM attribute (0018,9087).

Our study has several limitations. First, there were small, unbalanced datasets in the scout and perfusion, which may cause relatively low accuracy in the ML classification algorithm. However, if the dataset is accumulated and becomes large, this problem can be solved automatically. Indeed, we observed that the classification accuracy increased when adding the multi-center trial datasets, mainly because there were fewer incorrect answers due to learning from the combined dataset. Secondly, we trained our ImageSort-net using MRI data acquired from six MRI manufacturers, which may raise the issue of generalizability. However, these six manufacturers have more than 90% of the market share worldwide. In addition, our MRI datasets were acquired from a total of 25 hospitals, which enabled us to develop a generalizable MRI sequence classification algorithm.

## 5. Conclusions

In summary, the training of ML algorithms utilizing rule-based virtual labels has yielded high accuracy for sequence-type classification of brain MRI. This approach has empowered us to construct a robust, self-learning system with sustained efficacy. We believe that our innovative self-learning system plays a pivotal role in facilitating the seamless integration of changes and advancements in DICOM standards and the evolving landscape of MRI technologies. Future research endeavors should focus on further exploring the potential of developing AI algorithms centered on images leveraging ImageSort-net. Moreover, it remains imperative to investigate the feasibility of extending this methodology to encompass other modalities or anatomical structures.

## Figures and Tables

**Table 1:** Number of patients and MRI scans in datasets.

Dataset	Centers	Patients	MRI Scans	Manufacturers
Hospital dataset *	1	1528	1531	Philips (933), Siemens (507), GE (67), Hitachi (10), Toshiba (12), Medinus (2)
Multi-center trial dataset	8	59	256	Philips (57), Siemens (110), GE (87)

* Including 16 outside hospitals with 356 patients and 357 MRI scans.

**Table 2:** Extracted DICOM attribute in the preprocessing step.

	DICOM Attribute	DICOM Standard Attribute Description
Rule-based system	SeriesDescription	Description of the Series
	ProtocolName	User-defined description of the conditions under which the Series was performed.
Machine Learning	ScanningSequence	Description of the type of data taken.
	SequenceVariant	Variant of the scanning sequence.
	MRAcquisitionType	Identification of a spatial data encoding scheme.
	ImageType	Image identification characteristics.
	RepetitionTime	Time in ms between the beginning of a pulse sequence and the beginning of the succeeding (essentially identical) pulse sequence.
	EchoTime	Time in ms between the middle of the excitation pulse and the peak of the echo produced
	FlipAngle	Steady state angle in degrees to which the magnetic vector is flipped from the magnetic vector of the primary field.
	ImagingFrequency	Precession frequency in MHz of the nucleus being addressed
	NumberOfPhaseEncodingSteps	Total number of lines in k-space in the ‘y’ direction collected during acquisition.
	Rows	Number of rows in the image.
	Columns	Number of columns in the image.
	InversionTime	Time in msec after the middle of inverting the RF pulse to the middle of the excitation pulse to detect the amount of longitudinal magnetization.
	SliceThickness	Nominal slice thickness, in mm.
	ScanOption	Parameters of the scanning sequence.
	BodyPartExamined	Text description of the part of the body examined.
	ScliceNum *	The number of slices per series instance

* Not directly derived from the DICOM attribute.

**Table 3:** Sequence type classification performance of a rule-based labeling system.

Sequence Type	Hospital Dataset				Multi-Center Trial Dataset			
	Precision	Recall	F1-Score	No of Blanks *	Precision	Recall	F1-Score	No of Blanks *
T1	99.6%	99.5%	99.6%	329/4071	99.7%	95.1%	97.3%	10/420
T2	99.2%	99.6%	99.4%	14/1496	100%	99.3%	99.6%	0/146
FLAIR	99.8%	99.4%	99.6%	3/1422	99.2%	100%	99.6%	0/266
suscgre	99.7%	99.6%	99.6%	83/1752	99.7%	96.3%	98%	23/354
MRA	99.5%	99.6%	99.6%	962/3763	97.2%	99.4%	98.3%	258/1100
Scout	96.5%	92.6%	94.5%	13/308	98.4%	100%	99.2%	0/127
Perfusion	92.7%	100%	96.2%	4/55	86%	100%	92.5%	21/64
Diffusion	99.6%	100%	99.8%	667/3239	99.7%	99.8%	99.8%	35/669
Total ^†^	98.3%	98.8%	98.5%	2075/16,106	97.5%	98.7%	98%	347/3146

* The number of blanks indicates the number of series that the rule-based labeling system failed to infer (Number of blank/number of series type); ^†^ Total refers to the sum of all sequence types.

**Table 4:** Classification performance of ML_virtual_ and ML_human_ in the initial dataset (hospital dataset), subsequent dataset (multi-center trial dataset), and combined dataset (hospital + multi-center dataset).

Performances	ML_virtual_			ML_human_		
	Only Hospital Dataset	Only Multi-Center Trial Dataset	Combined Dataset *	Only Hospital Dataset	Only Multi-Center Trial Dataset	Combined Dataset *
Overall accuracy	98.5%	95.6%	99.7%	99%	99.4%	99.7%
Precision	88.6%	86.8%	99%	89.7%	98.2%	99%
Recall	86.1%	98.4%	99.9%	92.8%	99.8%	99.9%
F1-score	86.3%	90%	99.5%	90%	99%	99.5%

* Multi-center test dataset results.

**Table 5:** Performance of each sequence classification of ML_virtual_ and ML_human_ on each dataset.

	ML_virtual_			ML_human_		
	Precision	Recall	F1-Score	Precision	Recall	F1-Score
Initial dataset (hospital dataset only)						
Diffusion	100%	100%	100%	100%	100%	100%
FLAIR	66.6%	100%	80%	66.6%	100%	80%
MRA	98.4%	99.2%	98.8%	99.1%	99.6%	99.4%
Perfusion	100%	66.6%	80%	100%	100%	100%
Scout	46%	54.5%	50%	54%	70%	60%
suscgre	100%	92.2%	96%	100%	91.2%	95.4%
T1	97.8%	98.4%	98.1%	98.4%	99%	98.7%
T2	100%	77.8%	87.5%	100%	82.3%	90%
Subsequently added dataset (multi-center trial dataset only)						
Diffusion	100%	97.2%	98.6%	100%	100%	100%
FLAIR	-	-	-	-	-	-
MRA	100%	94.5%	97.3%	100%	99.2%	99.6%
Perfusion	95.2%	100%	97.5%	95.2%	100%	97.5%
Scout	-	-	-	-	-	-
suscgre	40%	100%	56.2%	95.6%	100%	97.7%
T1	100%	100%	100%	100%	100%	100%
T2	-	-	-	-	-	-
Combined dataset * (hospital dataset + multi-center trial dataset)						
Diffusion	100%	100%	100%	100%	100%	100%
FLAIR	-	-	-	-	-	-
MRA	100%	99.6%	99.8%	100%	99.6%	99.8%
Perfusion	95.2%	100%	97.5%	95.2%	100%	97.5%
Scout	-	-	-	-	-	-
suscgre	100%	100%	100%	100%	100%	100%
T1	100%	100%	100%	100%	100%	100%
T2	-	-	-	-	-	-

* Multi-center test dataset results.

## Data Availability

The datasets generated and analyzed during the current study are not publicly available due to the security of the data but are available from the corresponding author upon reasonable request.

## Supplementary Materials

The following supporting information can be downloaded at: https://www.mdpi.com/article/10.3390/diagnostics14010070/s1, Figure S1: Confusion matrix for sequence type classification performance of rule-based labeling system. a hospital dataset. b multi-center dataset.; Figure S2: Confusion matrix for sequence type classification performance in hospital dataset. a ML_virtual_ b ML_human_. Figure S3: Confusion matrix for sequence type classification performance in multi-center dataset. a ML_virtual_. b ML_human_. Figure S4: Multi-center test dataset classification results of models trained with combine dataset. a ML_virtual_. b ML_human_. Table S1: Number of SeriesDescription and ProtocolName attributes per sequence type in the hospital dataset.
