# Supplementary material for: Sequence-Type Classification of Brain MRI for Acute Stroke Using a Self-Supervised Machine Learning Algorithm
Source: Diagnostics (Basel). 2023 Dec 27;14(1):70. doi: 10.3390/diagnostics14010070 (PMC10804387; doi:10.3390/diagnostics14010070)
Supplement: Supplementary file 1 [file diagnostics-14-00070-s001.zip › diagnostics-2738860-supplementary.pdf]

Supplementary Materials

Supplementary Table S1. Number of SeriesDescription and ProtocolName attributes per sequence type in the hospital dataset

Sequence type	Series (GT)	Different types of SDs	Different types of PN
T1	4071	647	552
T2	1496	240	254
FLAIR	1422	152	187
Diffusion	3239	273	209
suscgre	1752	132	175
MRA	3763	447	342
Scout	308	137	66
Perfusion	55	41	38
Total*	16106	2070	1823

Note—GT = ground truth (i.e., human expert labeling), SD = SeriesDescription, PN = ProtocolName

*Total refers to sum of all sequence types

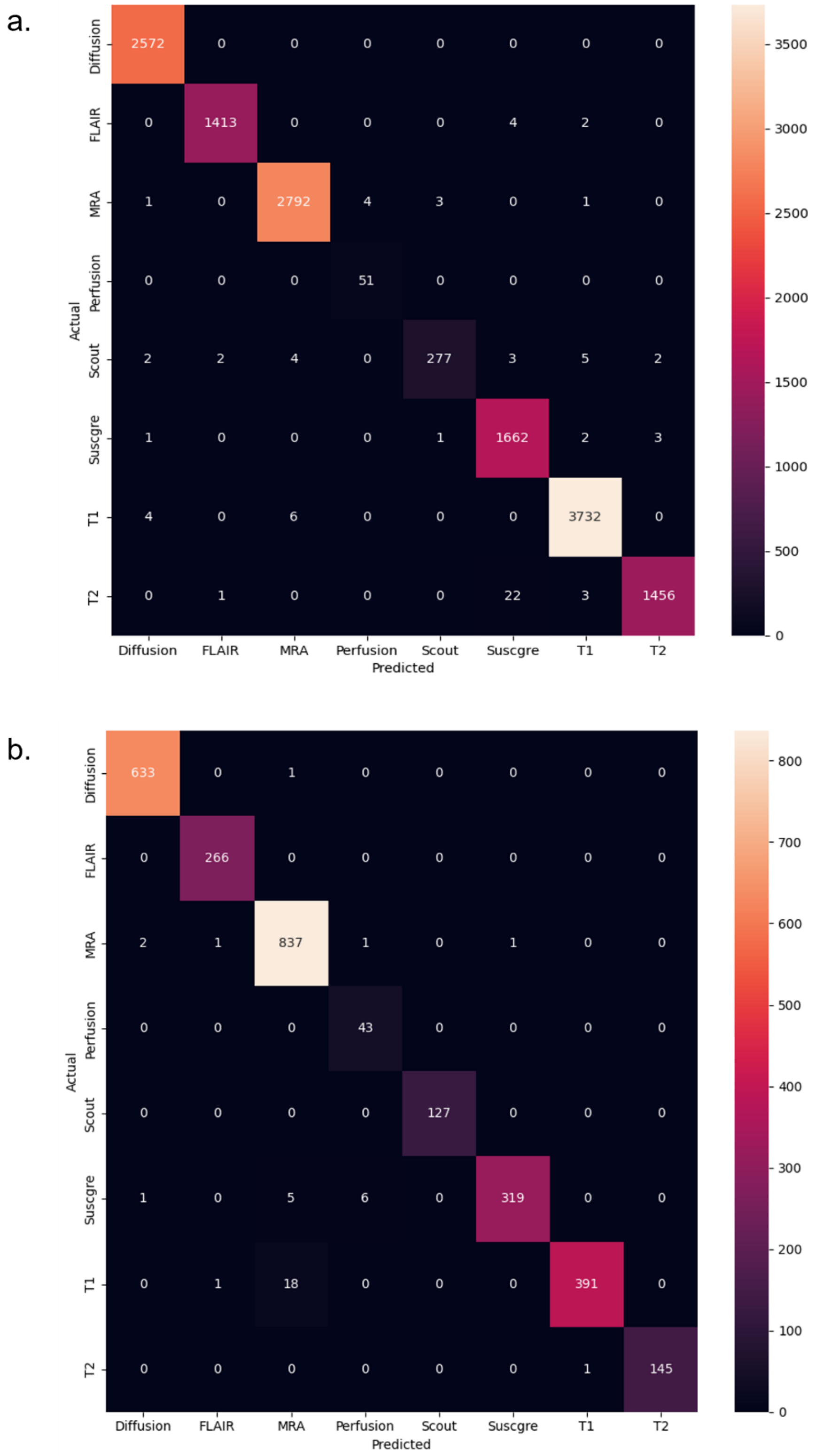

Supplementary Figure S1. Confusion matrix for sequence type classification performance of rule-based labeling system. **a** hospital dataset. **b** multi-center dataset.

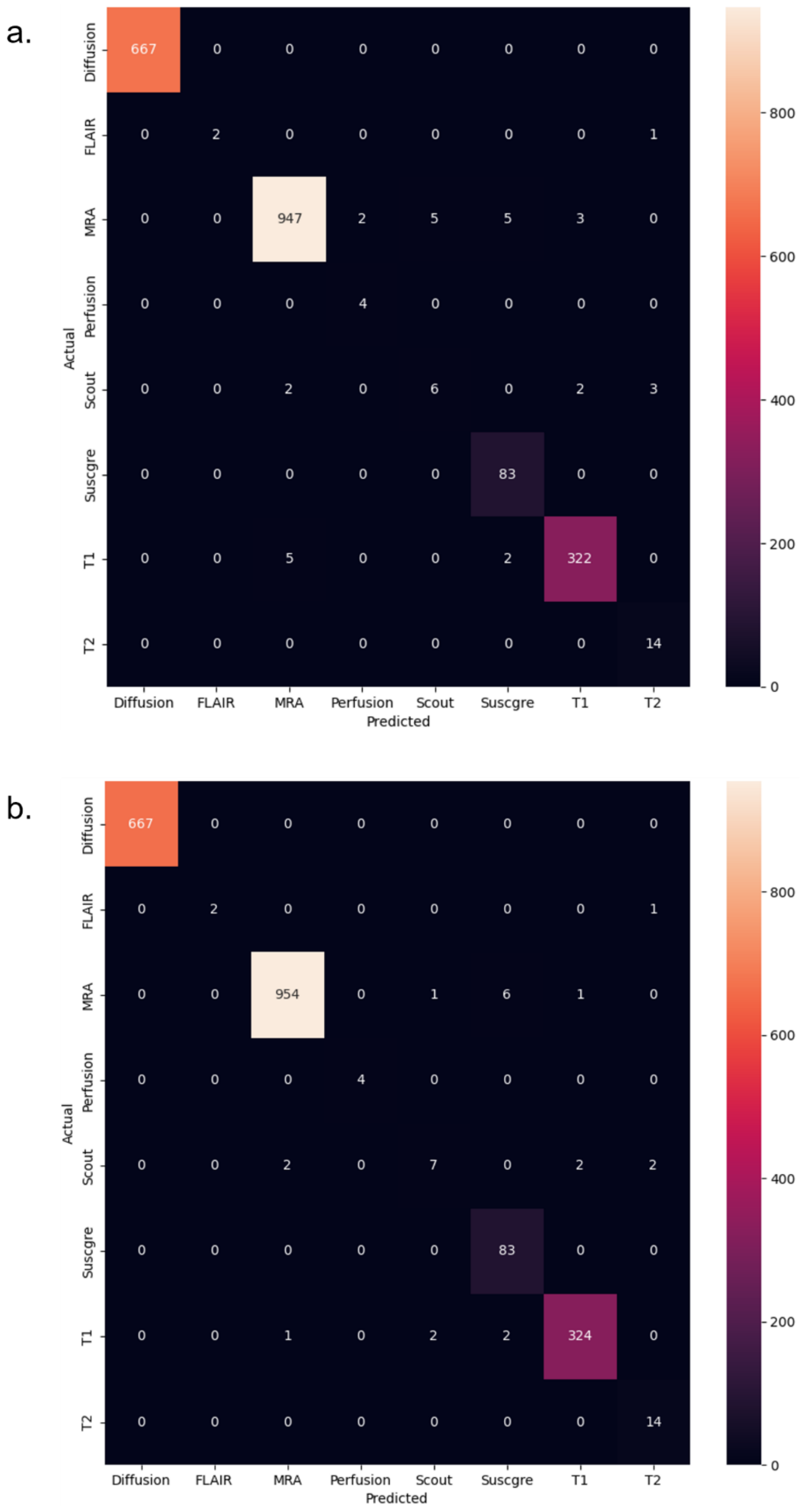

Supplementary Figure S2. Confusion matrix for sequence type classification performance in hospital dataset. **a** $ML_{virtual}$. **b** ML_{human}.

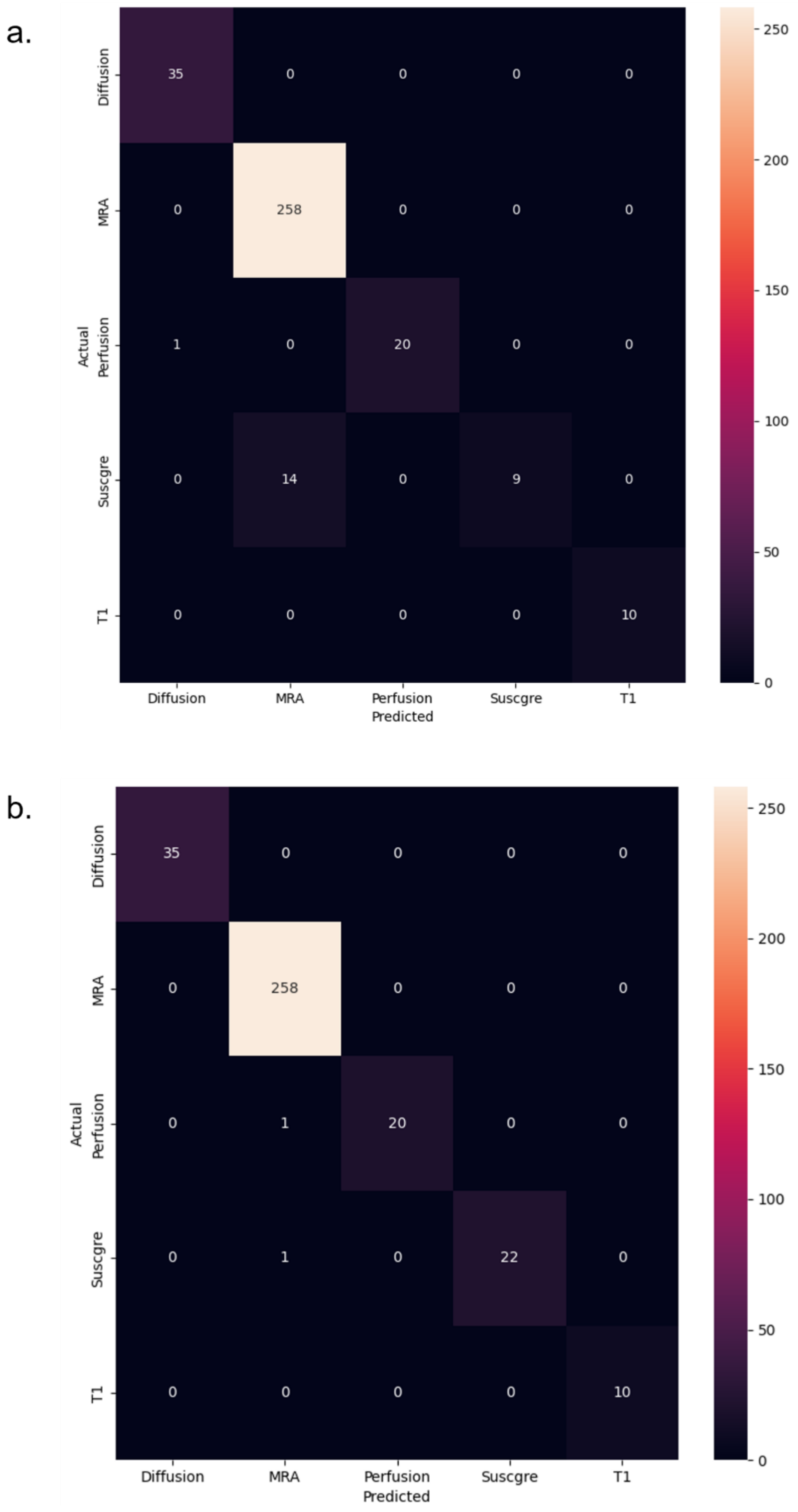

Supplementary Figure S3. Confusion matrix for sequence type classification performance in multi-center dataset. **a** $ML_{virtual}$. **b** ML_{human}.

a.

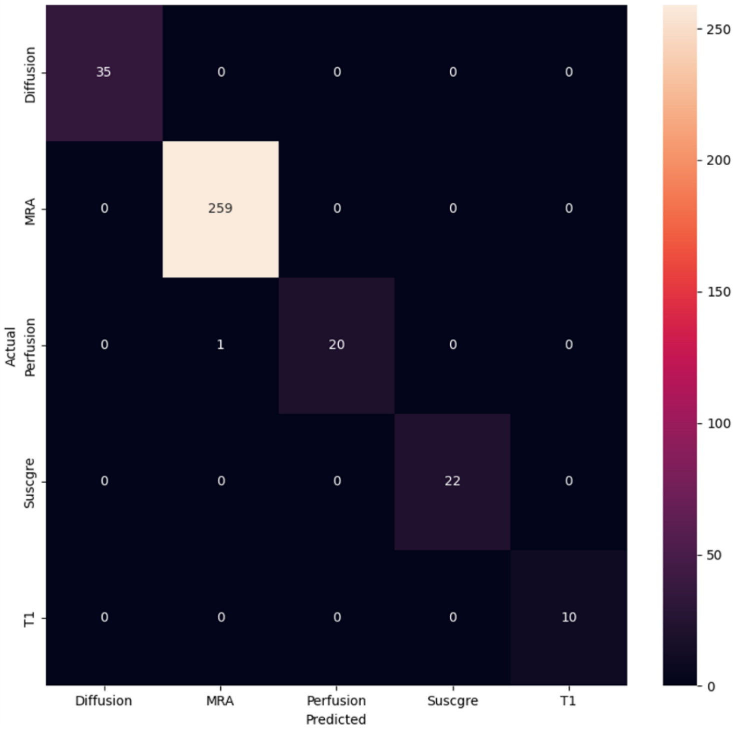

b.

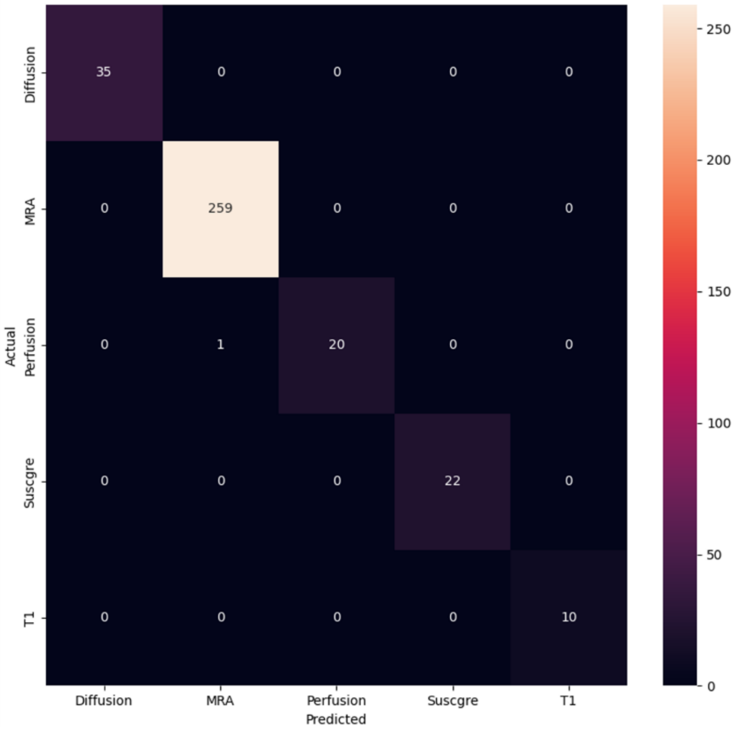

Supplementary Figure S4. Multi-center test dataset classification results of models trained with combine dataset. **a** $ML_{virtual}$. **b** ML_{human}.
